# A Novel α/β-Hydrolase Gene *IbMas* Enhances Salt Tolerance in Transgenic Sweetpotato

**DOI:** 10.1371/journal.pone.0115128

**Published:** 2014-12-12

**Authors:** Degao Liu, Lianjun Wang, Hong Zhai, Xuejin Song, Shaozhen He, Qingchang Liu

**Affiliations:** Beijing Key Laboratory of Crop Genetic Improvement/Laboratory of Crop Heterosis and Utilization, Ministry of Education, China Agricultural University, Beijing, China; National Taiwan University, Taiwan

## Abstract

Salt stress is one of the major environmental stresses in agriculture worldwide and affects crop productivity and quality. The development of crops with elevated levels of salt tolerance is therefore highly desirable. In the present study, a novel *maspardin* gene, named *IbMas*, was isolated from salt-tolerant sweetpotato (*Ipomoea batatas* (L.) Lam.) line ND98. IbMas contains maspardin domain and belongs to α/β-hydrolase superfamily. Expression of *IbMas* was up-regulated in sweetpotato under salt stress and ABA treatment. The *IbMas*-overexpressing sweetpotato (cv. Shangshu 19) plants exhibited significantly higher salt tolerance compared with the wild-type. Proline content was significantly increased, whereas malonaldehyde content was significantly decreased in the transgenic plants. The activities of superoxide dismutase (SOD) and photosynthesis were significantly enhanced in the transgenic plants. H_2_O_2_ was also found to be significantly less accumulated in the transgenic plants than in the wild-type. Overexpression of *IbMas* up-regulated the salt stress responsive genes, including pyrroline-5-carboxylate synthase, pyrroline-5-carboxylate reductase, *SOD*, *psbA* and phosphoribulokinase genes, under salt stress. These findings suggest that overexpression of *IbMas* enhances salt tolerance of the transgenic sweetpotato plants by regulating osmotic balance, protecting membrane integrity and photosynthesis and increasing reactive oxygen species scavenging capacity.

## Introduction

Soil salinization is becoming a serious threat to world agriculture to support a rapidly growing population [1], [2]. Approximately 20% of the irrigated soils in the world are under salt stress, and soil salinization has become a major constraint limiting crop production [3], [4]. The development of crops with elevated levels of salt tolerance is therefore highly desirable.

The superfamily of α/β-hydrolase fold enzymes is one of the largest known protein families, including hydrolases (acetylcholinesterase, carboxylesterase, dienelactone hydrolase, lipase, cutinase, thioesterase, serine carboxypeptidase, proline iminopeptidase, proline oligopeptidase, epoxide hydrolase) along with enzymes that require activation of HCN, H_2_O_2_ or O_2_ instead of H_2_O for the reaction mechanism (haloalkane dehalogenase, haloperoxidase, hydroxynitrile lyase) [5]–[8]. The ESTHER database, which is freely available via a web server (http://bioweb.ensam.inra.fr/esther) and is widely used, is dedicated to proteins with the α/β-hydrolase fold, and it currently contains>30 000 manually curated proteins [9]. The biological functions of α/β-hydrolase fold enzymes in various organisms are widely ranging and include biosynthesis, metabolism, signal transduction and gene regulation [8]. To date, a few of α/β-hydrolase fold enzymes such as esterase, phospholipase D and OsPOP5 have been shown to be involved in plant salt tolerance [10]–[13].

The maspardin (Mast syndrome, spastic paraplegia, autosomal recessive with dementia) protein is a member of the α/β-hydrolase superfamily. Maspardin was first identified as an intracellular binding protein for the cell surface glycoprotein CD4 and proposed to modulate CD4 stimulatory activity in humans [14], [15]. Simpson et al. [16] reported that the *maspardin* gene could cause the complicated form of hereditary spastic paraplegia known as Mast syndrome. However, the *maspardin* gene has not been characterized at the functional level in plants. In our previous study, an EST library was constructed by using salt-tolerant sweetpotato line ND98 and the suppression subtractive hybridization (SSH) technique, and it was found that expression of the *maspardin* gene, named *IbMas*, was significantly up-regulated in ND98 under salt stress (unpublished).

Sweetpotato, *Ipomoea batatas* (L.) Lam., is an important food and industrial material crop. It is also an alternative source of bio-energy as a raw material for fuel production [17]. The increased production of sweetpotato is desired, but this goal is often limited by salt stress [18]. Especially, sweetpotato as source of bio-energy will mainly be planted on marginal land. Salt stress is a critical delimiter for the cultivation expansion of sweetpotato. Therefore, the primary challenge facing scientists is enhancing sweetpotato's tolerance to salt stress to maintain productivity on marginal land. The improvement of this crop by conventional hybridization is limited because of its high male sterility, incompatibility and hexaploid nature [19]. Genetic engineering offers great potential to improve salt tolerance in this crop.

A number of genes have been isolated from sweetpotato based on the information gathered from related publications [18], [20]–[23]. However, very a little work has been done on the cloning of salt tolerance-associated genes in sweetpotato. Chen et al. [22] isolated *SPCP2* gene from sweetpotato and the *SPCP2*-overexpressing *Arabidopsis* plants exhibited higher salt and drought tolerance. Wang et al. [23] cloned *IbNFU1* gene from sweetpotato and the *IbNFU1*-overexpressing sweetpotato plants exhibited higher salt tolerance [24]. Liu et al. [18] cloned *IbP5CR* gene from sweetpotato and the *IbP5CR*-overexpressing sweetpotato plants exhibited higher salt tolerance. In this study, a novel *maspardin* gene, named *IbMas*, has been isolated from a salt-tolerant sweetpotato line ND98 and it is found that overexpression of *IbMas* gene can significantly enhance salt tolerance of the transgenic sweetpotato plants.

## Materials and Methods

### Plant materials

Salt-tolerant sweetpotato line ND98 was employed for gene cloning in this study. One expressed sequence tag (EST) clone was selected from the EST library of ND98 constructed at our laboratory, with 66.67% homology to a predicted *maspardin*-like gene (XP_004245715) from *Solanum lycopersicum* for cloning the gene. Sweetpotato cv. Shangshu 19, a commercial cultivar widely planted in China, was used for characterizing the function of the cloned gene in responses of the transgenic plants to salt stress.

### Cloning of *IbMas* gene

Total RNA was extracted from 0.5 g of fresh leaves of 4-week-old in vitro-grown plants of ND98 with the RNAprep Pure Plant Kit (Tiangen Biotech, Beijing, China). RNA samples were reverse-transcribed according to the instructions of Quantscript Reverse Transcriptase Kit (Tiangen Biotech, Beijing, China). A rapid amplification of cDNA ends (RACE) procedure was employed to amplify the 5′ and 3′ ends of the coding region using GeneRacer™ Kit (Invitrogen, Carlsbad, CA, USA). Based on the sequence of EST, primers were designed using the Primer 3 program (http://frodo.wi.mit.edu/primer3/) and listed in Table 1.

**Table 1:** Primers used in this study.

Primer name	Primer sequence (5′-3′)
Primers for 5′/3′ RACE	
*IbMas* 3′ RACE primer 1	TCTTGAAGACGGGAGGTGAT
*IbMas* 3′ RACE primer 2	GTTGAGGCACGGCCAGACTT
*IbMas* 5′ RACE primer 1	AAAATCACCTCCCGTCTTCA
*IbMas* 5′ RACE primer 2	TCCCGTCTTCAAGATTGCTT
Primers for constructing expression vector	
*IbMas*-oe-F	AGATCTATGAAAGGCGTCTTC
*IbMas*-oe-R	CACGTGCTATACTAAGTTTCTAAAT
Primers for identifying transformants	
35S-F	GAACTCGCCGTAAAGACTGG
*IbMas*-R	CCACTTTAGGGCCAAAATCA
Primers for real-time quantitative PCR	
*Actin*-F	AGCAGCATGAAGATTAAGGTTGTAGCAC
*Actin*-R	TGGAAAATTAGAAGCACTTCCTGTGAAC
*IbMas*-F	TGATTTTGGCCCTAAAGTGG
*IbMas*-R	AATCCACCGAGTGCTGTACC
*P5CR*-F	ATAGAGGCATTGGCTGATGG
*P5CR*-R	GGTAGTCCCACCTGGTGATG
*P5CS*-F	GCCTGATGCACTTGTTCAGA
*P5CS*-R	TTGAGCAATTCAGGGACCTC
*PRK*-F	GCTCTCAACATAGATCAGCT
*PRK*-R	TGAAGGCTCTACTATCTCAT
*psbA*-F	CATCCGTTGATGAATGGTTA
*psbA*-R	GCAACAGGAGCTGAGTATGC
*SOD*-F	TCCTGGACCTCATGGATTTC
*SOD*-R	GCCACTATGTTTCCCAGGTC

PCR amplifications were performed with an initial denaturation at 94°C for 3 min, followed by 35 cycles at 94°C for 30 s, 55°C for 30 s, 72°C for 1 min and final extension at 72°C for 10 min. PCR products were separated on a 1.0% (w/v) agarose gel. Target DNA bands were recovered by gel extraction, then cloned into PMD19-T (TaKaRa, Beijing, China), and finally transformed into competent cells of *Escherichia coli* strain DH5α. White colonies were checked by PCR and the positive colonies were sequenced (Invitrogen, Beijing, China).

### Sequence analysis of *IbMas* gene

The full-length cDNA of *IbMas* gene was analyzed by an online BLAST at the National Center for Biotechnology Information (NCBI) website (http://www.ncbi.nlm.nih.gov/). For the multiple sequence alignment analysis, the amino acid sequences of IbMas and other homologs from different plant species retrieved from NCBI were aligned using the DNAMAN software (Lynnon Biosoft, Quebec, Canada). The phylogenetic analysis was conducted with the Clustalx program. Theoretical molecular weight and isoelectric point (*p*I) were calculated using ProtParam tool (http://www.expasy.ch/tools/protparam.html). The conserved domain of IbMas protein was scanned by InterProScan program (http://www.ebi.ac.uk/interpro/).

### Expression analysis of *IbMas* gene

The expression of *IbMas* gene in ND98 was analyzed by real-time quantitative PCR (qRT-PCR) according to the method of Liu et al. [18]. The 4-week-old in vitro-grown plants of ND98 were submerged in 1/2 MS medium containing 200 mM NaCl and 100 µM abscisic acid (ABA), respectively, and sampled at 0, 3, 6, 12, 24 and 48 h after treatment to analyze the expression of *IbMas* gene. Specific primers of the *IbMas* gene were listed in Table 1. A 169 bp fragment of sweetpotato *β-actin* gene (Genbank AY905538), used as an internal control, was amplified by the specific primers (Table 1). Quantification of the gene expression was done with comparative *C*_T_ method [25].

### Transformation of sweetpotato with the *IbMas* gene

The coding region of *IbMas* gene was amplified from ND98 using a pair of specific primers with terminal *Bgl*II and *Pml*I restriction sites (Table 1) and then inserted into the same restriction sites in vector pCAMBIA3301 to create expression vector pCAMBIA3301-*IbMas* under the control of CaMV 35S promoter and NOS terminator of the expression box. This vector also contained *bar* gene driven by a CaMV 35S promoter. The recombinant vector was transformed into the *Agrobacterium tumefaciens* strain EHA 105 for sweetpotato transformation.

Embryogenic suspension cultures of sweetpotato cv. Shangshu 19 were prepared as described by Liu et al. [26]. Sixteen weeks after initiation, cell aggregates 0.7–1.3 mm in size from embryogenic suspension cultures of 3 days after subculture were employed for the transformation. The *IbMas*-overexpressing sweetpotato plants were produced according to the method of Liu et al. [18], but selection culture was conducted using 0.8 mg L^−1^ phosphinothricin (PPT).

### PCR analysis of the *IbMas*-overexpressing sweetpotato plants

PCR analysis of the putatively transgenic plants and wild-type plants was performed as described by Liu et al. [18]. Equal amounts of 200 ng of total DNA were amplified in 50 µL reactions using 35S forward and *IbMas*-specific reverse primers (Table 1). These primers were expected to give products of 640 bp. PCR amplifications were performed with an initial denaturation at 94°C for 3 min, followed by 35 cycles at 94°C for 30 s, 55°C for 30 s, 72°C for 1 min and final extension at 72°C for 10 min. PCR products were separated by electrophoresis on a 1.0% (w/v) agarose gel.

### In vitro assay for salt tolerance

Based on the method of He et al. [1], the transgenic plants and wild-type plants were cultured on Murashige and Skoog (MS) medium with 86 mM NaCl in order to evaluate their in vitro salt tolerance at 27±1°C under 13 h of cool-white fluorescent light at 54 µM m^−2^ s^−1^. Three plants were treated for each line. The growth and rooting ability were continuously observed for 4 weeks.

### Analyses of proline and MDA content, SOD activity and fresh weight

The transgenic plants and wild-type plants cultured on MS medium with 86 mM NaCl for 4 weeks were used to analyze their proline and malonaldehyde (MDA) content, superoxide dismutase (SOD) activity and fresh weight. Proline content and SOD activity were analyzed as described by He et al. [1]. MDA content was measured according to the method of Gao et al. [2]. The plant fresh weight was measured immediately.

### Expression analysis of *IbMas* gene in the transgenic plants

The expression of the *IbMas* gene in the transgenic plants and wild-type plants was analyzed by qRT-PCR. The transgenic and wild-type in vitro-grown plants were submerged for 12 h in 1/2 MS medium with 200 mM NaCl. qRT-PCR analysis was performed as described above.

### In vivo assay for salt tolerance

The transgenic plants and wild-type plants were transferred to soil in a greenhouse for further evaluation of salt tolerance. The cuttings about 25 cm in length were cultured in the Hoagland solution [27] with 0 and 86 mM NaCl, respectively. Three cuttings were treated for each line. The growth and rooting ability were continuously observed for 4 weeks.

The 25-cm-long cuttings of the salt-tolerant transgenic plants evaluated with water culture assay and wild-type plants were grown in 19-cm diameter pots containing a mixture of soil, vermiculite and humus (1∶1∶1, v/v/v) in a greenhouse, with one cutting per pot, for further assay for salt tolerance according to the method of Liu et al. [18].

### Measurement of photosynthesis

Photosynthetic rate, stomatal conductance and transpiration rate in the leaves of the salt-tolerant transgenic plants and wild-type plants grown in pots for 2 weeks under 200 mM NaCl stress were measured according to the methods of Liu et al. [18]. Relative chlorophyll content (SPAD value in fresh leaves) was measured as described by Fernández-Falcón et al. [28] with Chlorophyll Meter SPAD-502 (Minolta, Japan). The experiments were conducted at 9-11 a.m. of sunny days.

### Analysis of H_2_O_2_ accumulation

H_2_O_2_ accumulation in the leaves of the salt-tolerant transgenic plants and wild-type plants grown in pots for 2 weeks under 200 mM NaCl stress was analyzed by using 3, 3'-diaminobenzidine (DAB) staining as described by Liu et al. [18].

### Expression analyses of salt stress responsive genes

The expression of salt stress responsive genes in the salt-tolerant transgenic plants and wild-type plants was analyzed by qRT-PCR. The transgenic and wild-type in vitro-grown plants were submerged for 12 h in 1/2 MS medium containing 0 and 200 mM NaCl, respectively. The qRT-PCR analysis was performed as described by Liu et al. [18]. Specific primers designed from conserved regions of genes were listed in Table 1. Sweetpotato *β-actin* gene was used as an internal control (Table 1). Quantification of the gene expression was done with comparative *C*_T_ method [25].

### Statistical analysis

The experiments were repeated three times and the data presented as the mean ± SE were analyzed by Student's *t*-test in a two-tailed analysis to compare the parameters obtained under normal or salt stress conditions. A *P* value of <0.05 or <0.01 was considered to be statistically significant.

## Results

### Cloning and sequence analysis of *IbMas* gene

**Figure 1:**
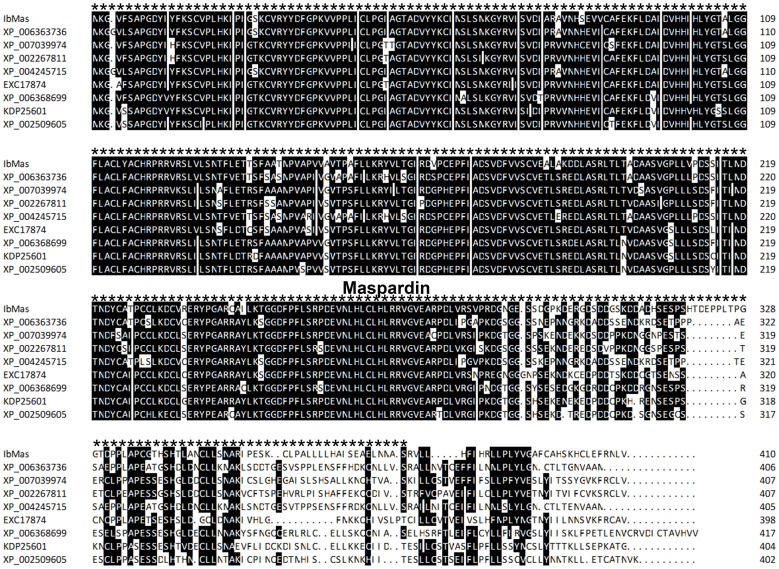
Sequence alignment of IbMas protein with its homologous proteins from various plant species. The proteins are as follows: XP_006363736 from *Solanum tuberosum*, XP_007039974 from *Theobroma cacao*, XP_002267811 from *Vitis vinifera*, XP_004245715 from *Solanum lycopersicum*, EXC17874 from *Morus notabilis*, XP_006368699 from *Populus trichocarpa*, KDP25601 from *Jatropha curcas* and XP_002509605 from *Ricinus communis*. The maspardin domain is marked by asterisk line.

**Figure 2:**
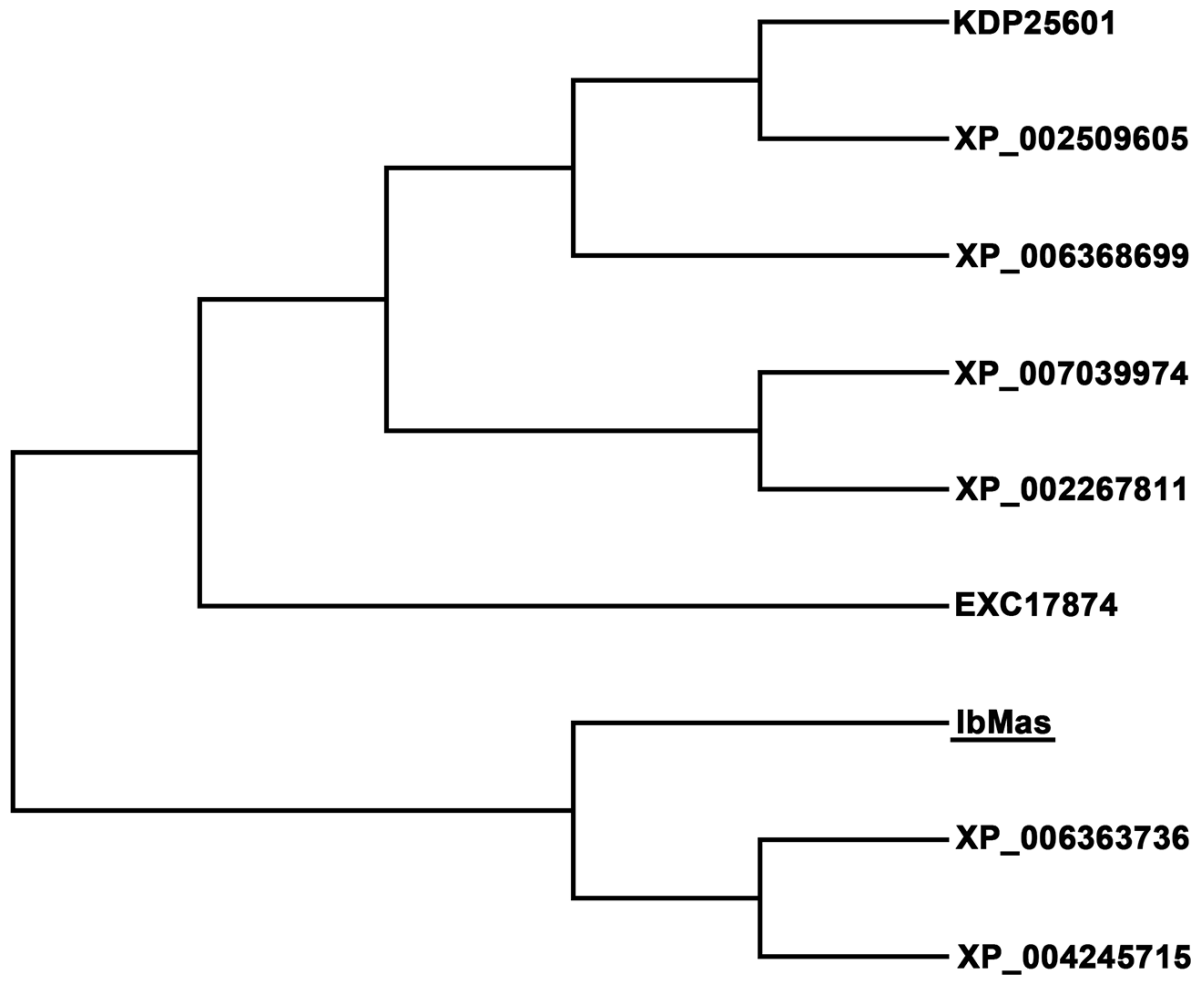
Phylogenetic tree of IbMas protein with its homologous proteins. The branch lengths are proportional to distance.

The *IbMas* gene was cloned from salt-tolerant sweetpotato line ND98 by RACE and submitted to GenBank (accession no. KM095957). The cDNA sequence of 1564 bp contained an 1233 bp open reading frame (ORF) encoding a 410 amino acids polypeptide with a molecular weight of 45.4 kDa and an isoelectric point (*p*I) of 6.27. Sequence analysis via InterPro program (http://www.ebi.ac.uk/interpro/) showed that IbMas protein contained typical maspardin domain and belonged to α/β-hydrolase superfamily (Fig. 1). A BLASTX search indicated that no homolog of known function was similar to IbMas in plants, while the amino acid sequence of IbMas showed 66.99% to 71.53% amino acid identity with predicted protein products of XP_002509605 from *Ricinus communis*, KDP25601 from *Jatropha curcas*, XP_006368699 from *Populus trichocarpa*, EXC17874 from *Morus notabilis*, XP_004245715 from *Solanum lycopersicum*, XP_002267811 from *Vitis vinifera*, XP_007039974 from *Theobroma cacao* and XP_006363736 from *Solanum tuberosum*. Phylogenetic analysis revealed that IbMas had a close relationship with predicted protein products of XP_006363736 from *Solanum tuberosum* and XP_004245715 from *Solanum lycopersicum* (Fig. 2).

### Expression analysis of *IbMas* gene in ND98

**Figure 3:**
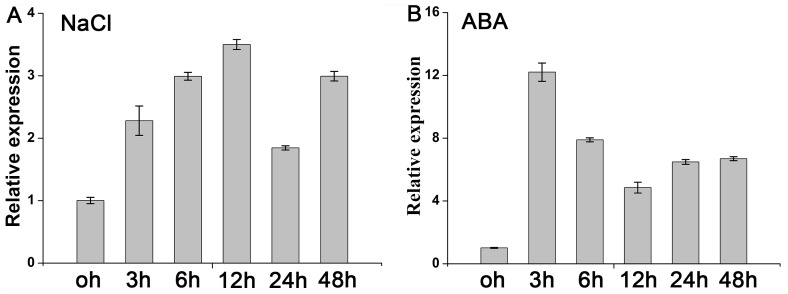
Expression analysis of the *IbMas* gene in sweetpotato line ND98 by real-time quantitative PCR. (A) and (B) Relative expression level of *IbMas* in ND98 after different times (h) of 200 mM NaCl and 100 µM ABA treatment, respectively. The 4-week-old in vitro-grown plants of ND98 were submerged in 1/2 MS medium containing 200 mM NaCl and 100 µM ABA, respectively, and sampled at 0, 3, 6, 12, 24 and 48 h after treatment to analyze the expression of *IbMas*. The sweetpotato *β-actin* gene was used as an internal control. Data are presented as means ± SE (n = 3).

The expression of *IbMas* gene in response to salt stress and ABA treatment was examined by qRT-PCR (Fig. 3). An increase in the *IbMas* transcript was observed after 3 h of exposure to 200 mM NaCl, peaked at 12 h with the 3.5-fold higher expression level than that of untreated control, and thereafter declined (Fig. 3A). For 100 µM ABA treatment, the expression of *IbMas* was induced rapidly to the highest level (12.2-fold) at 3 h, followed by a decrease (Fig. 3B).

### Production of the *IbMas*-overexpressing sweetpotato plants

**Figure 4:**
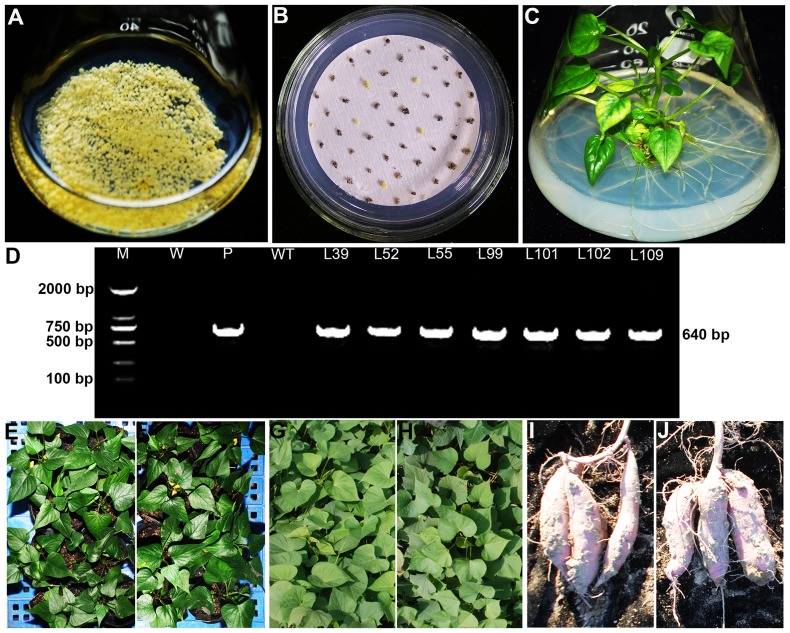
Production of transgenic sweetpotato plants overexpressing the *IbMas* gene. (A) Embryogenic suspension cultures rapidly proliferating in MS medium containing 2.0 mg L^−1^ 2,4-D. (B) PPT-resistant calluses formed on MS medium with 2.0 mg L^−1^ 2,4-D, 100 mg L^−1^ Carb and 0.8 mg L^−1^ PPT after 8 weeks of selection. (C) Regeneration of plantlets from PPT-resistant calluses on MS medium with 1.0 mg L^−1^ ABA, 100 mg L^−1^ Carb and 0.8 mg L^−1^ PPT. (D) PCR analysis of transgenic plants. Lane M: DL2000 DNA marker; Lane W: water as negative control; Lane P: plasmid pCAMBIA3301-*IbMas* as positive control; Lane WT: wild-type as negative control; Lanes L39, L52, L55, L99, L101, L102 and L109: transgenic plants. (E) and (F) WT and transgenic plants grown in a greenhouse, respectively. (G) and (H) WT and transgenic plants grown in a field, respectively. (I) and (J) Storage roots of WT and transgenic plants, respectively.

A total of 1000 cell aggregates of sweetpotato cv. Shangshu 19 (Fig. 4A) cocultivated with *A. tumefaciens* strain EHA 105 were cultured on the selective medium with 2.0 mg L^−1^ 2,4-dichlorophenoxyacetic acid (2,4-D), 100 mg L^−1^ carbenicillin (Carb) and 0.8 mg L^−1^ PPT. Eight weeks after selection, 72 PPT-resistant embryogenic calluses were produced from them (Fig. 4B) and transferred to MS medium with 1.0 mg L^−1^ ABA, 100 mg L^−1^ Carb and 0.8 mg L^−1^ PPT. After 5 to 6 weeks, 61 of them formed somatic embryos which further germinated into plantlets on the same medium (Fig. 4C). These plantlets developed into whole plants on the basal medium. A total of 173 putatively transgenic plants, named L1, L2, …, L173, respectively, were obtained in the present study.

The 173 putatively transgenic plants were analyzed by PCR amplification. The results showed that 119 of them had a specific 640 bp band of the *IbMas* gene, while no amplification occurred with the DNA from the remaining 54 putatively transgenic plants and wild-type plant and water (Fig. 4D), indicating that the 119 plants were transgenic.

### Improved salt tolerance in the *IbMas*-overexpressing sweetpotato

**Figure 5:**
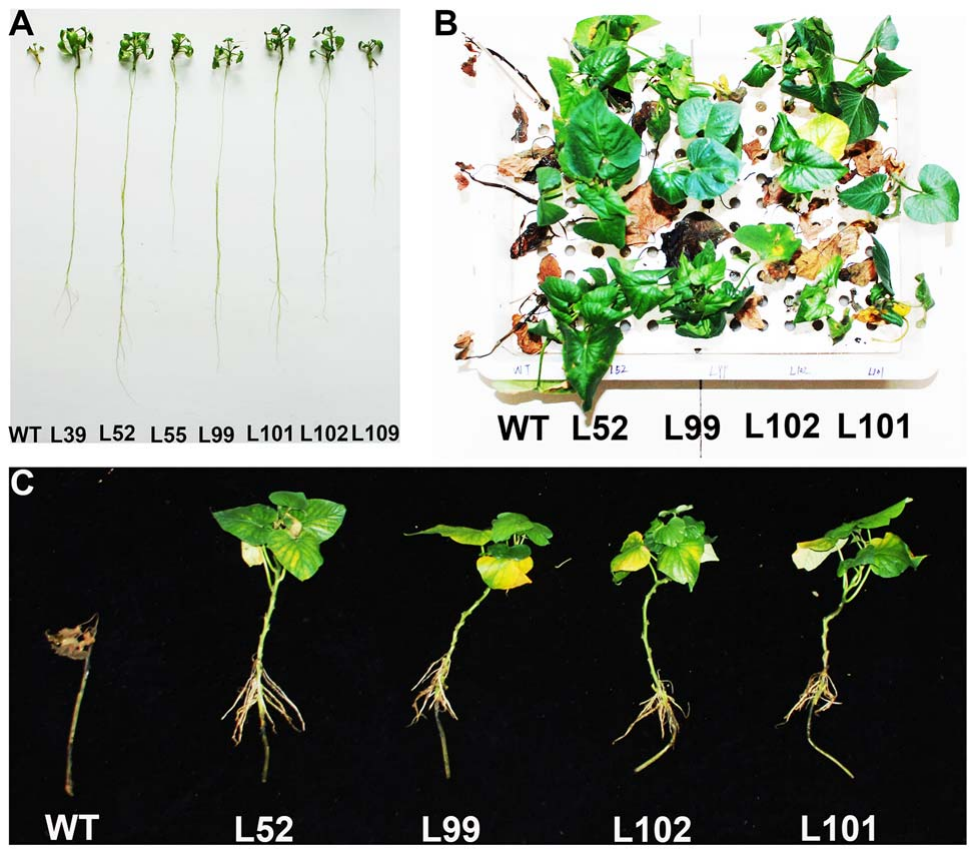
Responses of the *IbMas*-overexpressing sweetpotato plants under 86 mM NaCl stress. (A) The growth and rooting of trangenic plants and wild-type plant (WT) cultured for 4 weeks on MS medium supplemented with 86 mM NaCl. (B) and (C) Phenotypes of salt-tolerant transgenic plants (L52, L99, L102 and L101) and WT incubated for 4 weeks in Hoagland solution with 86 mM NaCl; all of the cuttings of L52, L99, L102 and L101 formed obvious new leaves and roots and those of WT died.

Thirty-six transgenic plants were randomly sampled for evaluating their salt tolerance. The transgenic plants and wild-type plants showed the similar growth and rooting on MS medium without NaCl. After cultured for 4 weeks on MS medium with 86 mM NaCl, the transgenic plants exhibited vigorous growth and good rooting in contrast to the poor-growing wild-type plants (Fig. 5A). This observation indicated that the transgenic plants had higher salt tolerance than wild-type plants.

Proline and MDA content, SOD activity and fresh weight of the 36 transgenic plants were shown in Table 2. Proline content, SOD activity and fresh weight were significantly higher in the 11 transgenic plants than in wild-type plants, while MDA content was significantly lower in these 11 transgenic plants than in wild-type plants. These results suggest that the high salt tolerance observed is due, at least in part, to the modulation of existing salt tolerance pathways.

**Table 2:** Comparison of salt tolerance between the *IbMas*-overexpressing plants and wild-type plants.

Plant lines	Proline content (µg g^−1^ FW)	SOD activity (U g^−1^ FW)	MDA content (nM g^−1^ FW)	Fresh weight (g^−1^ plant)
L52	53.76±1.25**^a^	564.88±6.77**	19.46±0.40**	1.16±0.03**
L99	52.58±2.21**	553.84±10.89**	19.17±0.54**	1.07±0.09**
L101	51.78±0.92**	533.59±12.33**	20.52±0.56**	0.98±0.03**
L102	49.47±1.22**	539.67±7.89**	20.77±1.35**	0.93±0.08**
L51	48.44±0.60**	532.13±6.68**	21.78±0.54**	0.82±0.04**
L39	48.01±1.30**	537.77±14.27**	21.70±0.98**	0.85±0.07**
L55	47.98±1.06**	501.66±6.78**	23.20±0.32**	0.69±0.06**
L28	47.64±0.93**	472.39±7.52*	29.92±0.55	0.56±0.05*
L37	46.06±0.58**	513.29±4.88**	22.62±0.69**	0.76±0.05**
L53	45.38±1.83**	503.51±3.98**	22.40±0.51**	0.71±0.05**
L59	45.00±1.71**	502.44±14.36*	26.05±1.35*	0.78±0.06**
L109	44.34±2.41*	496.79±8.04**	26.28±1.74*	0.60±0.04**
L3	43.38±2.75*	450.54±15.05	30.37±0.53	0.61±0.06**
L75	41.23±2.77*	478.03±3.75**	29.23±0.51	0.53±0.03**
L79	40.88±1.25**	489.78±10.54*	29.52±1.25	0.55±0.03**
L43	40.77±0.59**	453.31±7.05	30.40±0.58	0.51±0.04*
L17	38.02±2.32	440.66±11.44	30.41±0.63	0.47±0.04
L61	37.08±2.61	471.61±5.59*	26.98±0.38**	0.50±0.04*
L96	36.12±2.09	455.28±5.93	25.35±0.91**	0.47±0.02*
L11	35.97±2.98	493.68±3.51**	28.32±0.52*	0.49±0.02**
L23	35.08±1.84	463.01±7.82	25.78±1.11*	0.46±0.03*
L113	34.93±2.84	448.83±9.98	31.01±1.39	0.42±0.03
L88	33.81±1.28	432.31±14.33	31.46±0.79	0.44±0.03*
L72	33.60±1.74	454.37±3.02	30.92±1.52	0.45±0.05
L81	32.97±0.83	414.84±14.34	31.85±0.74	0.45±0.02*
L47	32.75±2.54	446.26±8.43	32.15±1.88	0.39±0.04
L9	32.51±1.28	467.60±12.04	30.90±0.64	0.41±0.04
WT	31.94±1.27	436.33±6.48	31.75±0.79	0.32±0.03
L69	31.62±0.98	428.81±5.21	31.68±1.71	0.40±0.04
L18	31.05±0.66	410.09±10.21	33.97±1.95	0.41±0.03
L92	30.68±1.05	418.41±5.68	32.30±1.48	0.38±0.03
L1	30.56±0.60	386.15±2.68	34.75±1.16	0.35±0.02
L60	30.26±1.33	424.87±8.12	33.94±0.59	0.34±0.03
L70	29.93±2.05	408.56±6.32	32.78±0.32	0.36±0.04
L83	29.86±0.85	396.41±2.51	35.35±1.19	0.32±0.03
L67	28.42±1.76	380.32±9.51	35.43±1.59	0.31±0.01
L10	27.96±1.86	395.35±4.04	35.08±0.87	0.31±0.02

a Data are presented as means ± SE (n = 3). * and ** indicate a significant difference from that of the wild-type (WT) at *P*<0.05 and <0.01, respectively, by Student's *t*-test.

**Figure 6:**
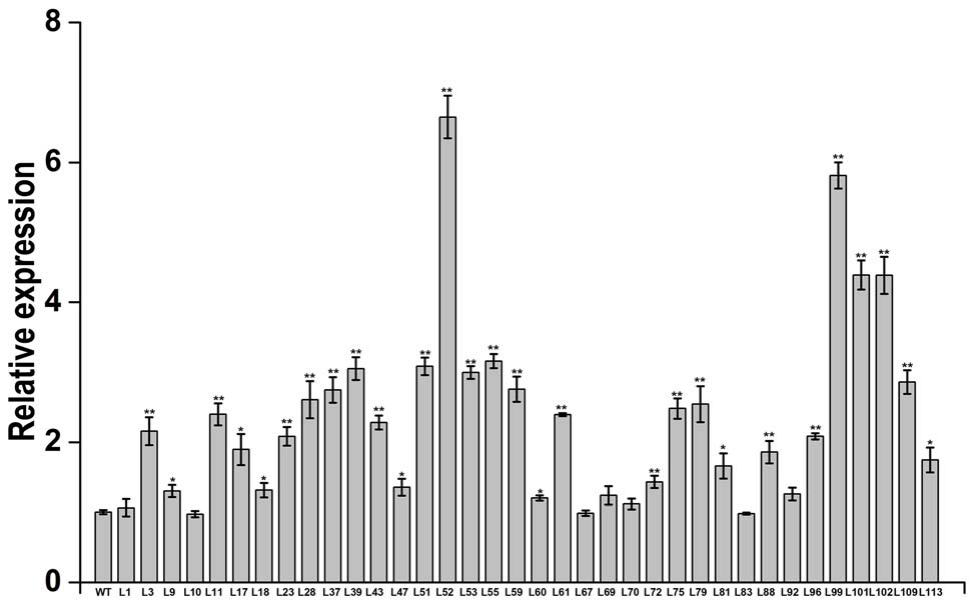
Expression analysis of *IbMas* gene in the transgenic sweetpotato plants by real-time quantitative PCR. The 36 transgenic and wild-type (WT) in vitro-grown plants were submerged in 1/2 MS medium with 200 mM NaCl for 12 h to analyze the expression of *IbMas*. The sweetpotato *β-actin* gene was used as an internal control. The results are expressed as relative values based on WT as reference sample set to 1.0. Data are presented as means ± SE (n = 3). * and ** indicate a significant difference from that of WT at *P*<0.05 and <0.01, respectively, by Student's *t*-test.

qRT-PCR analysis indicated that there was positive correlationship between expression level of *IbMas* gene and salt tolerance of transgenic plants (Fig. 6). Especially, the 4 transgenic plants, L52, L99, L101 and L102, showed significantly higher level of *IbMas* gene expression than that of the other 32 transgenic plants and wild-type (Fig. 6).

The 11 transgenic plants and wild-type plants were transferred to the soil in a greenhouse and a field, and showed 100% survival (Fig. 4E-J). No morphological variations were observed (Fig. 4E-J). For further evaluation of salt tolerance, the cuttings of these 11 transgenic plants and wild-type plants were cultured for 4 weeks in the Hoagland solution containing 0 and 86 mM NaCl, respectively. The growth and rooting of all cuttings were normal without NaCl. And at 86 mM NaCl, the 4 transgenic plants (L52, L99, L101 and L102) formed obvious new leaves and roots, the number and length of which were significantly higher compared to wild-type plants; the 5 transgenic plants survived, but failed to form new leaves; the 2 transgenic plants and wild-type plants gradually turned brown to death (Fig. 5B, C; Table 3). These results demonstrated that L52, L99, L101 and L102 had significantly higher salt tolerance than the other transgenic plants and wild-type plants.

**Table 3:** Leaf and root formation of the *IbMas*-overexpressing sweetpotato plants after 4 weeks of water culture with 86 mM NaCl.

Plant lines	Leaf formation	No. of roots	Length of roots (cm)
L52	++^a^	29.33±3.84**^b^	7.03±1.23**
L99	++	25.00±5.13*	7.07±1.19**
L102	++	23.67±4.48*	5.83±0.60**
L101	++	19.33±2.33**	6±1.65*
L37	+	14.67±4.98	4.83±0.17**
L39	+	14.33±2.96*	4.97±2.01
L53	+	13.00±5.69	3.23±1.82
L51	+	10.00±3.79	4.53±1.54
L55	+	8.67±2.33	4.23±2.12
L109	-	4.33±0.67	3.50±1.44
L59	-	4.00±1.53	2.83±1.27
WT	-	3.33±0.88	1.03±0.12

a ‘++’ indicates that cuttings formed obvious new leaves; ‘+’ indicates that cuttings survived, but failed to form new leaves; ‘-’ indicates that cuttings died.

b Data are presented as means ± SE (n = 3). * and ** indicate a significant difference from that of the wild-type (WT) at *P*<0.05 and <0.01, respectively, by Student's *t*-test.

**Figure 7:**
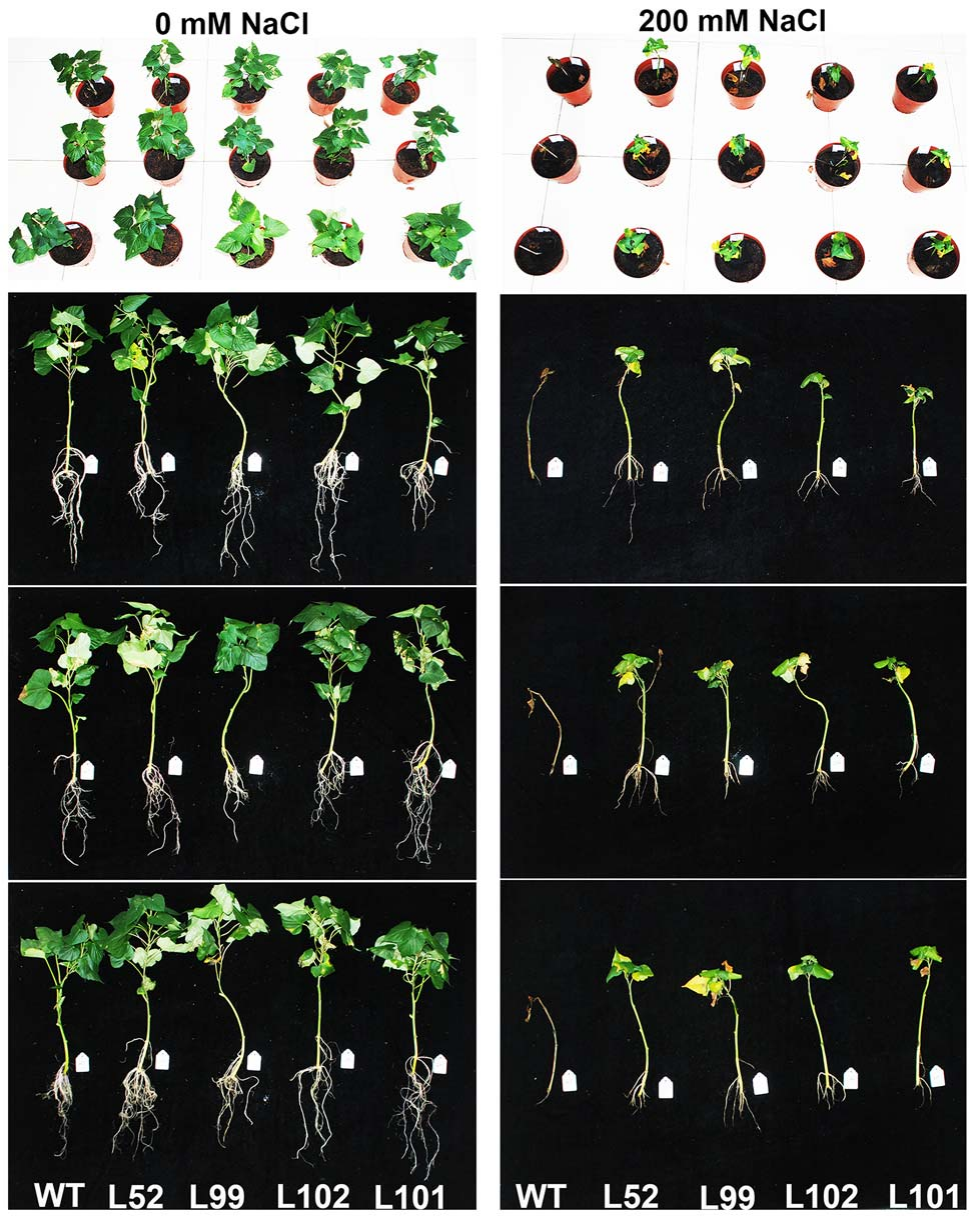
Phenotypes of the *IbMas*-overexpressing sweetpotato plants grown in pots under 200 mM NaCl stress. The 25-cm-long cuttings of the salt-tolerant transgenic plants (L52, L99, L102 and L101) and wild-type plants (WT) were grown in 19-cm diameter pots containing a mixture of soil, vermiculite and humus (1∶1∶1, v/v/v) in a greenhouse, with one cutting per pot. All pots were irrigated sufficiently with half-Hoagland solution for 10 days until the cuttings formed new leaves, and then each pot was irrigated a 200 mL of 0 and 200 mM NaCl solution, respectively, once every 2 days for 4 weeks. All of L52, L99, L102 and L101 plants showed good growth and increased physical size and those of WT died under 200 mM NaCl stress.

**Figure 8:**
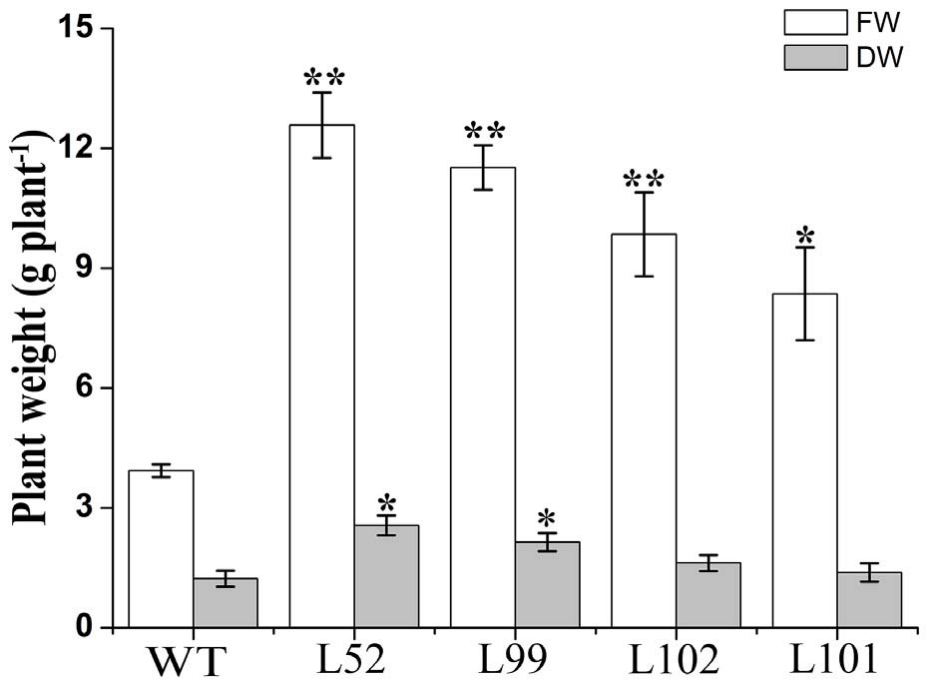
Biomass of the *IbMas*-overexpressing sweetpotato plants grown in pots under 200 mM NaCl stress. The 25-cm-long cuttings of the salt-tolerant transgenic plants (L52, L99, L102 and L101) and WT were grown in 19-cm diameter pots containing a mixture of soil, vermiculite and humus (1∶1∶1, v/v/v) in a greenhouse, with one cutting per pot, and treated as described in Fig. 6. After treatment, the plant fresh weight (FW) was measured immediately. The plants were then dried for 24 h in an oven at 80°C and weighed (DW). All treatments were performed in triplicate. Data are presented as means ± SE (n = 3). * and ** indicate a significant difference from that of WT at *P*<0.05 and <0.01, respectively, by Student's *t*-test.

The 4 salt-tolerant transgenic plants L52, L99, L101 and L102 and wild-type plants were grown in pots and irrigated a 200 mL of 0 and 200 mM NaCl solution, respectively, once every 2 days for 4 weeks. The growth and rooting of all cuttings were normal without NaCl (Fig. 7). And at 200 mM NaCl, the 4 salt-tolerant plants showed good growth and increased physical size, while wild-type plants died (Fig. 7). Fresh weight (FW) and dry weight (DW) of the 4 salt-tolerant plants were increased by 113–220% and 12–108%, respectively, compared to the wild-type (Fig. 8).

### Enhanced photosynthesis in the salt-tolerant transgenic plants

**Figure 9:**
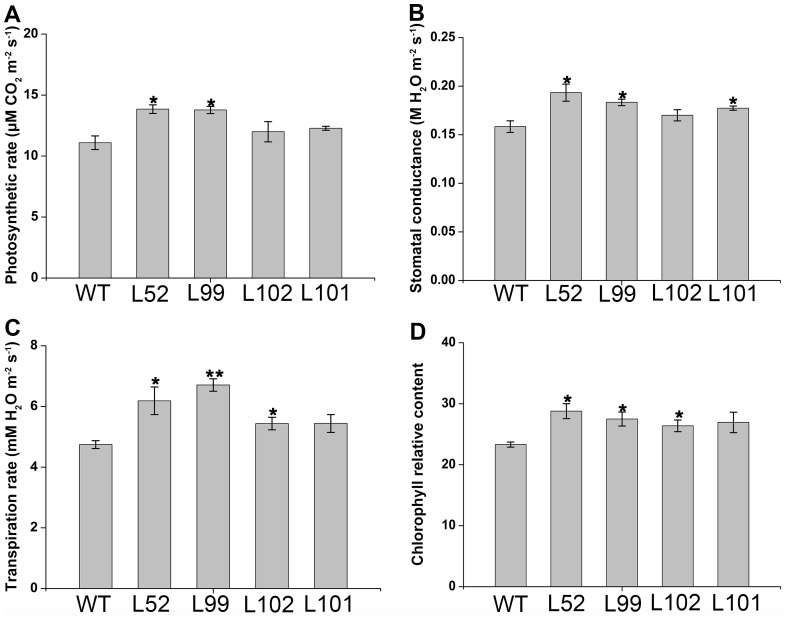
Photosynthetic performance of the *IbMas*-overexpressing sweetpotato plants under salt stress. (A), (B), (C) and (D) Photosynthetic rate, stomatal conductance, transpiration rate and chlorophyll relative content, respectively, in the leaves of salt-tolerant transgenic plants (L52, L99, L102 and L101) and wild-type plant (WT). The 25-cm-long cuttings of the salt-tolerant transgenic plants evaluated with water culture assay and WT were grown in 19-cm diameter pots containing a mixture of soil, vermiculite and humus (1∶1∶1, v/v/v) in a greenhouse, with one cutting per pot. All pots were irrigated sufficiently with half-Hoagland solution for 10 days until the cuttings formed new leaves, and then each pot was irrigated with a 200 mL of 200 mM NaCl solution once every 2 days for 2 weeks. Data are presented as means ± SE (n = 3). * and ** indicate a significant difference from that of WT at *P*<0.05 and <0.01, respectively, by Student's *t*-test.

Photosynthesis in the leaves of the 4 salt-tolerant transgenic plants grown in pots for 2 weeks under 200 mM NaCl stress was measured. The salt-tolerant transgenic plants maintained significantly higher photosynthetic rate, stomatal conductance, transpiration rate and chlorophyll relative content, which were increased by 8–25%, 7–22%, 15–41% and 13–24%, respectively, compared to the wild-type (Fig. 9).

### Reduced H_2_O_2_ accumulation in the salt-tolerant transgenic plants

**Figure 10:**
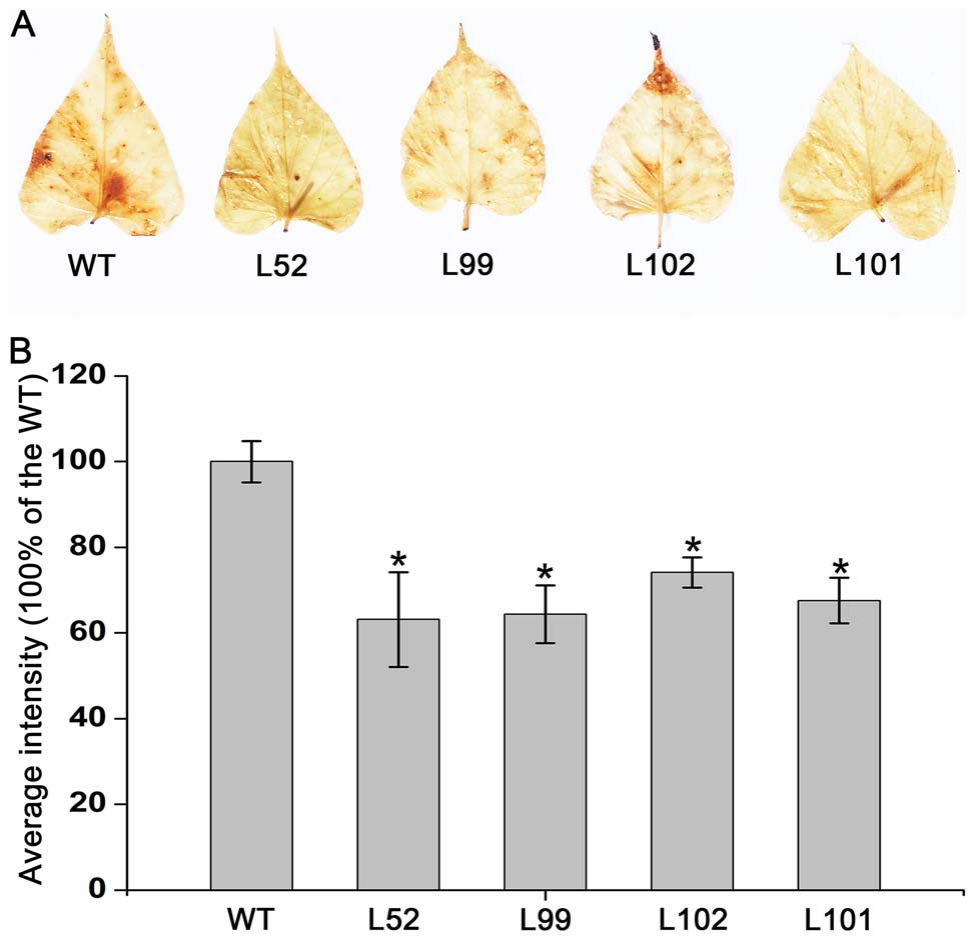
Effects of salt stress on H_2_O_2_ accumulation in the *IbMas*-overexpressing sweetpotato plants. (A) Accumulation of H_2_O_2_ in the leaves of salt-tolerant transgenic plants (L52, L99, L102 and L101) and wild-type plant (WT). A plant grown in a 19-cm diameter pot was irrigated with a 200 mL of 200 mM NaCl solution once every 2 days for 2 weeks. (B) The average intensity of DAB staining leaves after converting to 256 grey scale images. Data are presented as means ± SE (n = 3). * and ** indicate a significant difference from that of WT at *P*<0.05 and <0.01, respectively, by Student's *t*-test.

Abiotic stress induces the accumulation of H_2_O_2_, which is the toxic molecule that causes oxidative damage in plants [29]. To explore the potential mechanism by which *IbMas* improved salt tolerance in sweetpotato, H_2_O_2_ accumulation was analyzed by using DAB staining of leaves from the 4 salt-tolerant transgenic plants and wild-type plants under 200 mM NaCl stress for 2 weeks. The leaves of the salt-tolerant transgenic plants displayed less brown spots and diffuse staining than those of wild-type plants, indicating less H_2_O_2_ accumulation in the salt-tolerant transgenic plants (Fig. 10A). The statistical analysis further confirmed that significantly less H_2_O_2_ was accumulated in the salt-tolerant transgenic plants compared to the wild-type under salt stress (Fig. 10B).

### Expression analyses of salt stress responsive genes

**Figure 11:**
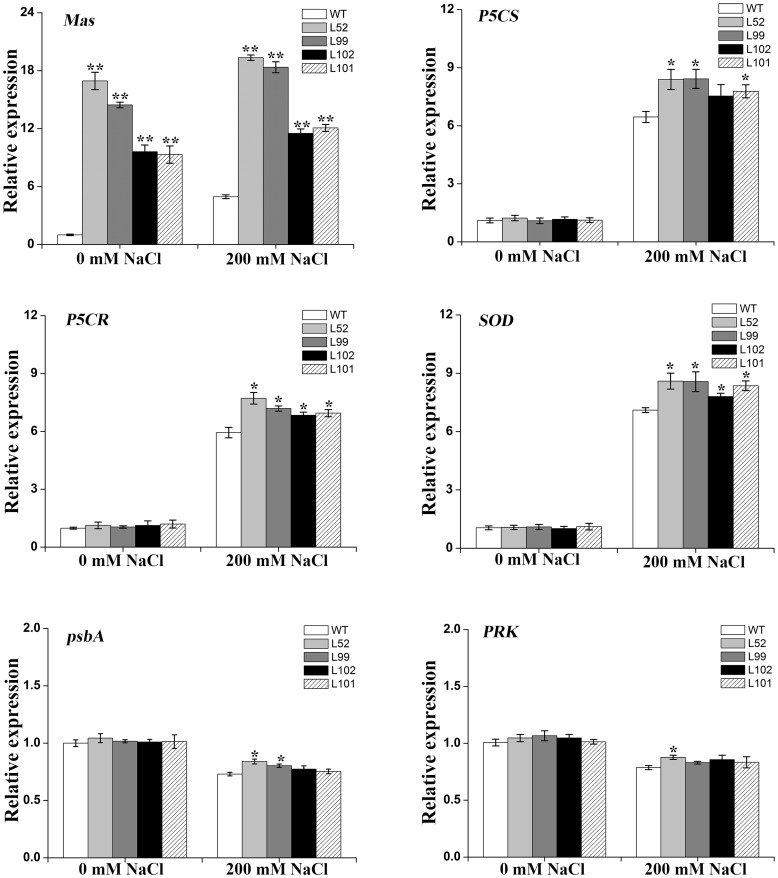
Relative expression level of *IbMas* and salt stress responsive genes in the *IbMas*-overexpressing sweetpotato plants. *P5CS*: pyrroline-5-carboxylate synthase; *P5CR*: pyrroline-5-carboxylate reductase; *SOD*: superoxide dismutase; *psbA*: encoding D1 protein; *PRK*: phosphoribulokinase (PRKase). The salt-tolerant transgenic plants (L52, L99, L102 and L101) and wild-type plant (WT) in vitro-grown plants were submerged for 12 h in 1/2 MS medium containing 0 and 200 mM NaCl, respectively. The sweetpotato *β-actin* gene was used as an internal control. The results are expressed as relative values based on WT grown under control condition as reference sample set to 1.0. Data are presented as means ± SE (n = 3). * and ** indicate a significant difference from that of WT at *P*<0.05 and <0.01, respectively, by Student's *t*-test.

Expression of *IbMas*, proline biosynthesis, photosynthesis and *SOD* genes in the salt-tolerant transgenic plants was analyzed by qRT-PCR. The expression level of *IbMas* gene was significantly higher in the transgenic plants compared to wild-type plants (Fig. 11). To investigate the impact of *IbMas* overexpression on the transcription of salt stress response related genes, the expression of well-known salt stress responsive marker genes encoding pyrroline-5-carboxylate synthase (P5CS), pyrroline-5-carboxylate reductase (P5CR) and SOD was analyzed under salt stress (Fig. 11). *P5CS*, *P5CR* and *SOD* genes exhibited significantly increased expression level in the salt-tolerant transgenic plants compared to the wild-type under salt stress (Fig. 11). The expression level of *psbA* and *PRK* genes, which encode D1 protein and phosphoribulokinase (PRKase), respectively, was also higher in transgenic plants than in wild-type plants (Fig. 11).

## Discussion

Soil salinity is one of the major factors that limit the productivity and quality of crops. Plant genetic engineering provides the potential for breeding salt-tolerant varieties. Overexpression of salt tolerance related genes is an important strategy for improving salt tolerance of crops.

The maspardin protein was first identified as an intracellular binding protein for the cell surface glycoprotein CD4 and proposed to modulate CD4 stimulatory activity [14]. Simpson et al. [16] reported that a nucleotide insertion (601insA) mutation in the *maspardin* gene resulted in the complicated form of hereditary spastic paraplegia known as Mast syndrome. However, the *maspardin* gene has not been characterized at the functional level in plants. In the present study, we isolated the *maspardin* gene from salt-tolerant sweetpotato line ND98. Sequence analysis showed that the protein contained typical maspardin domain and thus is named IbMas (Fig. 1). BLAST analysis indicated that IbMas protein exhibited 62.68% amino acid identity to an α/β-hydrolase superfamily protein in *Arabidopsis thaliana* (NP_192960). However, there was no report about *Arabidopsis* mutant for NP_192960. Furthermore, the present results demonstrated that no homolog of known function in other plants was similar to IbMas. Therefore, it is thought that *IbMas* is a novel gene.

The expression of *IbMas* gene was induced by salt stress and peaked at 12 h of salt stress (Fig. 3A), indicating that *IbMas* may play an important role in response of sweetpotato to salt stress. We found that overexpression of *IbMas* significantly enhanced the salt tolerance of sweetpotato (Figs. 5, 7). In addition, *IbMas* was also induced in the presence of ABA, and the transcript reached the highest level at 3 h under ABA treatment (Fig. 3B). It is well established that salt stress is able to induce ABA biosynthesis and trigger ABA-dependent signaling pathways [30], [31]. Thus, it is assumed that *Ibmas* gene may regulate sweetpotato salt stress response in an ABA-dependent manner similar to those of *OsMYB2* in rice [32], *AtLPK1* in *Arabidopsis* [33] and *LcDREB2* in *Leymus chinensis* [34].

Osmotic stress often results in more accumulation of proline, and the level of proline accumulation is related to the extent of salt tolerance [18], [24], [35], [36]. In the present study, most of the *IbMas*-overexpressing sweetpotato plants had significantly higher proline content compared to wild-type plants under salt stress, indicating measurable improvement of salt tolerance (Table 2; Figs. 5, 7). Proline accumulation in the *IbMas*-overexpressing sweetpotato plants most likely maintains the osmotic balance between the intracellular and extracellular environment under salt stress, which results in the improved salt tolerance [37], [38]. Also, proline helps cells to maintain membrane integrity [39], [40] and has been proposed to function as molecular chaperone stabilizing the structure of proteins [41]. Therefore, it is assumed that proline accumulation in the *IbMas*-overexpressing sweetpotato plants might protect the cell membrane from salt-induced injuries. It was also found that the expression of *P5CS* and *P5CR* genes was up-regulated in the transgenic sweetpotato plants under salt stress (Fig. 11). Thus, the present results suggest that overexpression of *IbMas* in sweetpotato plants increases proline accumulation by up-regulating the expression of *P5CS* and *P5CR* genes.

MDA is often considered a reflection of cellular membrane degradation, and its accumulation increases with production of superoxide radicals and hydrogen peroxide [29]. Higher MDA content can induce cell membrane damage, which further reduces salt tolerance of plants [18], [24], [42], [43]. In the present study, most of the *IbMas*-overexpressing sweetpotato plants had significantly lower MDA content compared to wild-type plants, also indicating the marked improvement of their salt tolerance (Table 2).

Salinity perturbs plant water uptake in leaves, leading to quick response in stomatal conductance. It also disrupts the osmotic, ionic and nutrient balances in plants. This affects photosynthetic electron transport and the activities of enzymes for carbon fixation [44], [45]. Salinity induces ROS production in plant cells [29], [46], [47]. It is thought that an effect of ROS is the inhibition of the repair of photodamaged PSII by the suppression of de novo protein synthesis; the primary sites of photodamage are the oxygen-evolving complex and the D1 proteins [48]. The damaging effects of singlet oxygen and hydroxyl radicals on PSII can be reduced by proline in isolated thylakoid membranes [49]. Proline protects PSII photofunctions against photodamage which gets accelerated in plants under salt stress [49], [50], [51]. In our study, the *IbMas*-overexpressing sweetpotato plants exhibited higher photosynthetic rate, stomatal conductance, transpiration rate and chlorophyll relative content compared to wild-type plants under salt stress (Fig. 9). Also, the expression of *psbA* and *PRK* genes was up-regulated in the transgenic plants (Fig. 11). The biomass difference between the *IbMas*-overexpressing plants and wild-type plants is thought to be due to the photosynthesis difference under salt stress (Fig. 8). The less affected photosynthesis of the *IbMas-*overexpressing sweetpotato plants could be explained by that the accumulated proline in the transgenic plants provides protection against photoinhibition under salt stress.

Salinity leads to the overproduction of reactive oxygen species (ROS) in plants which are highly reactive and toxic and cause damage to proteins, lipids, carbohydrates and DNA which ultimately results in oxidative stress. ROS scavenging systems of plants detoxify ROS to minimize and/or prevent oxidative damage in cells by increasing the activity of ROS scavenging enzymes [52]. As a key enzyme of ROS scavenging system, SOD is usually induced by salinity to enhance the timely dismutation of superoxide into oxygen and H_2_O_2_, which is subsequently removed through different pathways [53], [54]. Thus, SOD activity is often used to test the salt tolerance of plants [18], [24], [55], [56]. In the present study, the *IbMas*-overexpressing sweetpotato plants had significantly higher SOD activity and significantly less H_2_O_2_ accumulation compared to wild-type plants, which further showed the marked improvement of their salt tolerance (Table 2; Fig. 10). Consistent with this phenomenon, the increased *SOD* expression was also detected in the transgenic plants (Fig. 11). It is suggested that the improved salt tolerance of the transgenic sweetpotato plants is also due to the enhanced ROS scavenging capacity [18], [24], [54], [55], [56]. It has been reported that proline acted as a ROS scavenger under abiotic stress [57]. Proline is an effective scavenger of singlet oxygen and hydroxyl radicals [57], [58]. Thus, our results support that more proline accumulation in the *IbMas*-overexpressing sweetpotato plants increases the expression of *SOD* gene, which enhances ROS scavenging capacity.

In conclusion, a novel *maspardin* gene, *IbMas*, has been successfully isolated from salt-tolerant sweetpotato line ND98. The *IbMas*-overexpressing sweetpotato plants exhibited significantly higher salt tolerance compared with the wild-type. Our results suggest that overexpression of *IbMas* enhances salt tolerance of the transgenic sweetpotato plants by regulating osmotic balance, protecting membrane integrity and photosynthesis and increasing reactive oxygen species scavenging capacity.
